# Bias caused by a popular weighting scheme

**DOI:** 10.1107/S1600576724011889

**Published:** 2025-02-01

**Authors:** Julian Henn

**Affiliations:** a DataQ Intelligence UG, Fichtelgebirgsstrasse 66, Germany; Universität Duisburg-Essen, Germany

**Keywords:** data-quality metrics, data-quality evaluation, systematic errors, weighting schemes, weights

## Abstract

It is reported how the popular *SHELXL*-like weighting scheme leads to bias in residuals when the standard uncertainty (s.u.) values of the observed intensities are underestimated, which appears to be the case very frequently. An underestimation of the s.u. values by a factor of 5 destroys the symmetry of the residuals with respect to zero and leads to an average increase in *U*_equiv_ of 3.35% in a simulation.

## Abstract 2

The systematic error of systematically too small formal standard uncertainties of the observed intensities cannot be corrected for by the popular *SHELXL*-like weighting scheme. Instead, it disguises the error and induces bias in the equivalent isotropic atomic displacement parameters.

## 1. Introduction

Wilson (1976 ▸) advocates using weights *w* with the property 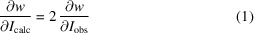
to avoid statistical bias in least-squares refinement against observed intensities. Any smooth and differentiable function of (*I*_obs_ − 2*I*_calc_) conforms to this requirement. However, equation (1) was derived under several approximations that limit its applicability. For example, the weights are expanded in a power series of the statistical fluctuation ε of a particular observed intensity (and of the deviation δ of the calculated intensity from the true value) only to the second order, which is justified only if ε (and δ) is small. For very weak intensities, however, the statistical fluctuation may be dominant. For this reason, Wilson states: ‘Third-order and higher terms have been neglected and these may be of importance for the very weak reflections.’ In the popular *SHELXL* refinement software package (Sheldrick, 2015 ▸), the condition (1) is implemented in the weighting scheme in the form of the parameter 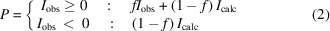
with *f* = 1/3 as standard, such that *I*_obs_ ≥ 0 follows *P* = 1/3 *I*_obs_ + 2/3 *I*_calc_, which obviously conforms to the above requirement as
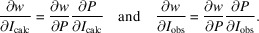
For negative intensity observations, however, equation (2) is discontinuous and does not conform to equation (1) anymore.

Regardless of the exact definition of *P*, a bias is introduced by weights conforming to equation (1), as the same absolute unweighed residual ±|Δ| may lead to vastly different weighted residuals ±*w*|Δ| depending on the sign of Δ. For an accidentally positive fluctuation of Δ = +|*I*_obs_| for a weak reflection with true value close to or identical to zero and *I*_calc_ = 0, as an example, *P* = *I*_obs_/3, whereas the same absolute value but with negative fluctuation Δ = −|*I*_obs_| leads to *P* = 0, which makes the absolute negative weighted residual larger than the corresponding positive weighted residual. Thus, weights in accordance with equation (1) down-weight positive fluctuations and up-weight negative fluctuations specifically of weak intensities. The symmetry of the weighted residuals with respect to positive and negative fluctuations is broken, which may lead to a significant negative shift of the weighted residuals, as will be seen later.

## 2. Application

The above-discussed bias is particularly strong when the standard uncertainties of the observed reflections after data integration, s.u.(*I*_obs_), severely underestimate the physical noise in the data. The full form of the weighting scheme as implemented in *SHELXL* is defined according to 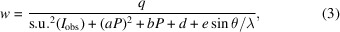
with parameters *a*, *b*, *c*, *d*, *e* and *f*. When the weighting-scheme parameter *c* is chosen to be 0, *q* is automatically set to 1. For *c* ≠ 0, exponential weights according to Dunitz & Seiler (1973 ▸) are used, with 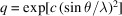 for *c* > 0 and 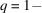

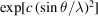
 for *c* < 0. For the overwhelming majority of all published datasets, *c* = 0 and only the parameters *a*, *b* and typically *f* = 1/3 are used. The need to use the parameter *b* > 0 is often taken as a warning sign. In the following, the focus is, for simplicity, on the case with *c* = *d* = *e* = 0 and *f* = 1/3, such that equation (3) simplifies to 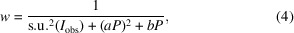
with *P* according to equation (2) and weighting-scheme parameters *a* and *b*.

Now suppose that the unknown true physical noise in the observed reflections is larger by the same factor *x* > 1 for all reflections,
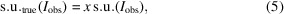
and that apart from this underestimation of the s.u.(*I*_obs_) there are no other systematic errors. What are the consequences?

The weak intensities will be affected most by this error, as their scatter is much more pronounced around zero as expected, thereby leading to large and highly significant negative intensity observations, *I*_obs_/s.u.(*I*_obs_) < −3. The high significance, however, is in this specific example of course only formal, as the s.u.(*I*_obs_) values are systematically underestimated. This leads to large positive and negative residuals, *i.e.* to a large variance in the residuals, specifically for the weak intensities. The weighting-scheme parameter *b* will account for these large variances for weak reflections. For the weak intensities with accidentally positive and negative fluctuations, the above-described bias will lead to strong negative residuals (as expected) but to damped positive residuals for the same absolute unweighted residual ±|Δ| with opposite sign. This effect might become so strong that it even affects the mean value of the whole distribution of weighted residuals by shifting it significantly to negative values. For a very simple example, think about the respective weighted residual 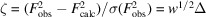 for opposite statistical fluctuations: assume s.u.(*I*_obs_) = 10, *I*_true, 1_ = *I*_calc, 1_ = *I*_true, 2_ = *I*_calc, 2_ = 0, *I*_obs, 1_ = 50 and *I*_obs, 2_ = −50, *i.e.* two reflections with the same true value of zero and the same s.u.(*I*_obs_), but with opposite statistical fluctuations that are larger than s.u.(*I*_obs_) by the same factor of 5 in both cases. The absolute values of the weighted residuals, ζ_1_ = *w*^1/2^Δ_1_ = +50/[10^2^ + *b*(50/3)]^1/2^ < +5 and ζ_2_ = *w*^1/2^Δ_2_ = −5, become quite different. For *b* = 10, as an example, ζ_1_ = +3.06 and ζ_2_ = −5.00.

### 2.1. Simulation

To explore further implications, two simulations with artificial data were conducted. First, the idealized case of an error-free experiment was simulated in order to construct a reference set and to confirm the validity of the simulation procedure (Sim 1). In the second simulation, a dataset was constructed following exactly the same protocol with only one small, but important, change. This change was to build in on purpose an artificial error in order to study its effect on the residuals. This artificial error increased the standard deviation of the Gaussian random number for each reflection by a factor of 5 when adding noise to the ideal data, such that the simulated physical noise in the simulated observed intensities was five times larger compared with the formal value s.u.(*I*_obs_) in the reflection input file (Sim 2). Some crystallographic values for these simulations are given in Table 1 ▸.

A small-molecule dataset (Shraddha *et al.*, 2020 ▸) was picked randomly from open access datasets published with *IUCrData* in the time range between 2020 and 2022. Details of the structure, crystallizing in the monoclinic space group *P*2_1_/*n* and chemically belonging to the imidazoles (C_29_H_23_ClN_2_O), and of a measurement taken at 297 K on a Bruker diffractometer with Mo *K*α radiation are to be found there. For the simulation, the refinement was repeated with the additional command OMIT −100 to incorporate all reflections including negative intensity observations in the refinement without modification. The calculated intensities were extracted from the resulting fcf file and used for the simulation by adding a Gaussian random number with a mean value of zero and standard deviation according to s.u.(*I*_obs_). This resulted in a consistent set of ideal simulated Bragg intensities and corresponding model parameters. The appropriately scaled simulated intensities and s.u.(*I*_obs_) values were written to a SHELX.hkl input file. In order to control the validity of the procedure, the model was refined against the simulated data. The simulation conforms to a high-quality structure refinement with respect to the given metrics: the weighting-scheme parameter values are both small and the weighted agreement factor *wR*(*F*^2^) = 0.0385 is also small, as is the goodness of fit (GoF) at 1.04. This simulation is, however, not entirely free of errors as the refinement still leads to very small but non-zero weighting-scheme parameters *a* = 0.0017 and *b* = 0.0047. It is expected that repeating the simulation procedure iteratively would lead to zero weighting-scheme parameters; however, the simulation was regarded as good enough for the purpose. The corresponding figures and numbers can be found in the supporting information. To prevent the software from subtracting anomalous signals from the simulated intensities (corresponding to idealized Bragg intensities free of anomalous dispersion), the individual atomic contributions to dispersion were set to zero with the help of the DISP command in the *SHELXL* input file.

In a second simulation, the same procedure was executed but the noise added was magnified by a factor of 5. The sole systematic error in the data is that the actual noise in the reflections is five times larger than the formal value. This has, however, severe effects on the weighting-scheme parameters and the residuals. Regarding the weighting-scheme parameters, the large value of *b* = 13.6794 in this case just indicates that the s.u.(*I*_obs_) values are all too small. That a large value of *b* may just indicate too small s.u.(*I*_obs_) is rarely discussed in the literature. A remedy for this situation in which no essential structure model deficiencies are present but large values of *b* appear is found in the online tutorial *Some thoughts on SHELXL weights* by Dr Parkin (https://xray.uky.edu/Tutorials/tutorials.html).

Table 2 ▸ shows some numbers describing the residuals for the reference simulation (Sim 1) and the simulation with systematic error (Sim 2). First we look at Sim 1. The mean value of the weighted residuals is virtually zero with an insignificant [〈ζ〉 = −0.12σ(〈ζ〉)] small negative deviation from zero. The mean values of the positive and absolute negative residuals are at 0.80 equal. The corresponding 3σ error bars are at 0.04 small and also equal. The mean values of the squared positive (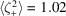) and squared negative residuals (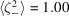) are close to one and within 3σ error bars (0.09 in both cases) identical, as expected for a high-quality refinement. There are 27 more negative than positive residuals, which corresponds to a ratio of 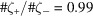. The small number of negative excess residuals is, however, not significant [*#*ζ_+_ − *#*ζ_−_ = −0.39(*N*_obs_)^1/2^]. In total, the residual distribution from simulation 1 is characterized by being symmetric with respect to positive and negative deviations, as expected for a dataset with little systematic error.

Now, we turn our attention to Sim 2. The mean value of the weighted residuals is shifted to −0.20, which is highly significant [〈ζ〉 = −8.00σ(〈ζ〉)]. The mean values of the positive and absolute negative residuals now differ substantially and cannot be regarded as equal within 3σ errors. The absolute value of the negative residuals has increased, with 〈|ζ_−_|〉 = 1.33 much stronger compared with the positive residuals (〈ζ_+_〉 = 0.83). The squared negative residuals are with 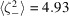 on average more than four times larger than the squared positive residuals (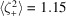). The number of positive residuals has increased so much that the excess number of positive residuals (227) is at 3.27(*N*_obs_)^1/2^ highly significant. In summary, the residual distribution of simulation 2 is characterized by being highly asymmetric with respect to positive and negative weighted residuals.

**Figure 1:**
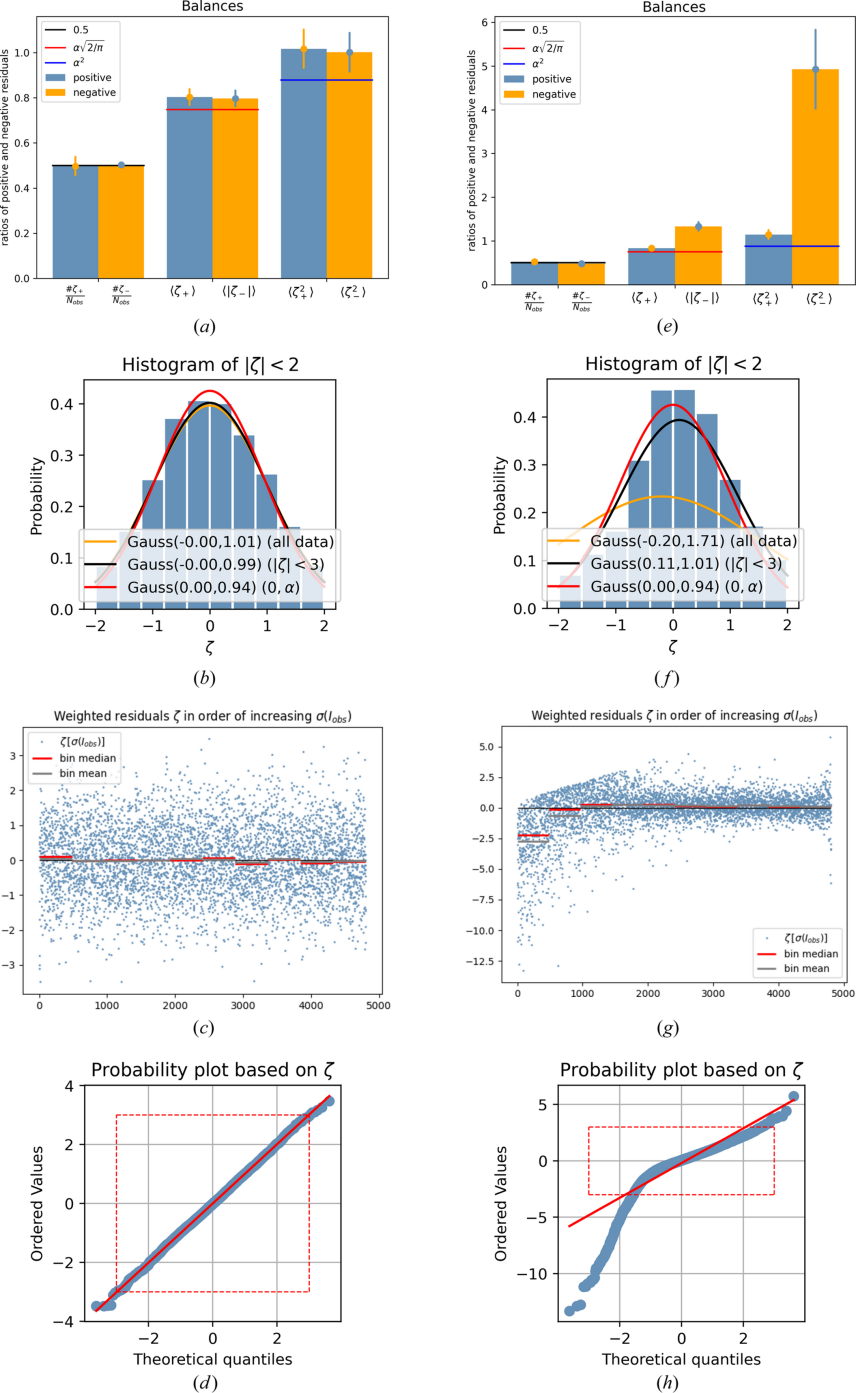
Diagnostic plots for Sim 1 (left) and Sim 2 (right). Row 1: ‘balance sheet’ with positive (blue) and negative (orange) residuals. First pair of columns: fraction of positive and negative residuals; second pair: mean value of just positive residuals and mean value of absolute negative residuals; and third pair: mean value of squared positive residuals and mean value of squared negative residuals. Additionally, 3σ error bars are attached. Row 2: histograms of weighted residuals with three Gaussian probability distributions – red: idealized; black: fitted to all absolute residuals smaller than 3, |ζ| < 3; and orange: fitted to all residuals. Row 3: weighted residuals printed in rank order of σ = 1/*w*^1/2^. Row 4: normal probability plots (Abrahams & Keve, 1971 ▸).

Fig. 1 ▸ visualizes the broken symmetry of the residuals. Whereas for simulation 1 the fractions of positive and negative residuals are virtually equal [Fig. 1 ▸(*a*), left] and positive and absolute negative mean residuals are similar [Fig. 1 ▸(*a*), middle], as are the mean squared positive and negative residuals [Fig. 1 ▸(*a*), right], the picture changes drastically for simulation 2, which shows stronger mean absolute negative residuals [Fig. 1 ▸(*e*), middle]. That the squared negative residuals are so much stronger indicates that large negative residuals play an important role [Fig. 1 ▸(*e*), right]. This might sound trivial, but it is not, as increased mean absolute negative residuals could also be induced by a shift of the residual distribution to negative values as a whole, which would be visible in a significant negative value 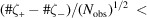

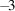
; however, Table 2 ▸ indicates a significant positive shift of 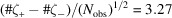. These seemingly contradicting findings are interpreted as follows: the least-squares procedure reacts to one-sidedly very large negative residuals by shifting the whole residual distribution as much as possible into the positive realm. Fig. 1 ▸(*f*) shows a histogram of the weighted residuals in a range between −2 and 2 for Sim 2. The red curve represents the probability density function for the ideal case, a Gaussian distribution with a mean value of zero and standard deviation 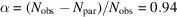. When a Gaussian probability density function is fitted to the subset of residuals |ζ| < 3, the parameters obtained are μ = +0.11 and σ = 1.01, *i.e.* a Gaussian shifted to positive values. When a Gaussian function is fitted to all weighted residuals ζ instead, the parameters obtained are μ = −0.20 and σ = 1.71, *i.e.* the total shift to negative residuals is clearly caused solely by the large residuals |ζ| > 3. Very importantly, Fig. 1 ▸(*g*) shows that the large negative residuals all originate from low values of σ(*I*_obs_) = 1/*w*^1/2^ (large weights, corresponding to weak intensities). As the chosen specific systematic error affects the weak intensities relatively most, large positive and negative residuals specifically for weak intensities are indeed expected. However, the positive residuals are simultaneously suppressed in the same region [Fig. 1 ▸(*g*)], which is a direct consequence of *b* > 0. This leads to a distinct asymmetry of the weighted residuals for large weights (corresponding to weak intensities). This pronounced asymmetry in the residuals for weak reflections leads to a shift of the median of the weighted residuals to negative values [see the red horizontal bars in Fig. 1 ▸(*g*)]. This additional shift is a direct consequence of the definition of *P* in combination with *b* > 0. It applies regardless of choosing *P* = *f* max(0, *I*_obs_) + (1 − *f*)*I*_calc_ or *P* = *f* *I*_obs_ + (1 − *f*)*I*_calc_ with all *I*_obs_ including negative intensity observations.

Before proceeding with a discussion, it is important to realize that there are two different but connected symmetry-breaking mechanisms at work. First, choosing *P* in accordance with equation (1) or equation (2) breaks the symmetry of the weighted residuals with respect to random positive and negative fluctuations for weak intensities. This results in a shift of the median of the weighted residuals of the weak intensities to negative values. This is a consequence of down-weighting accidentally positive fluctuations for weak intensities and up-weighting accidental negative fluctuations via P. Second, the actually expected large positive and negative weighted residuals for weak intensities are limited only for large positive weighted residuals by invoking *b* > 0.^1^ This is a consequence of choosing a weighting scheme of the type in equation (4).

It is particularly problematic that the large positive residuals are suppressed when it is additionally considered that large negative residuals are usually suppressed too, via the default choice of the OMIT command. The default is applied when the software user does not specify any OMIT command. Using the default OMIT command for Sim 2 leads after refinement to 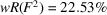 and GoF = 1.186, both much smaller compared with the refinement with all negative intensities included [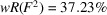 and GoF = 1.77, see Table 1 ▸]. The systematic error of s.u.(*I*_obs_) values that are much too small is masked by limiting large positive residuals via the weighting-scheme parameter *b* > 0 and limiting large negative residuals by omitting large negative fluctuations, which are, however, in this case, a valuable hint to the systematic error.

Finally, Fig. 1 ▸(*h*) shows how the normal probability plot is affected by this specific systematic error. The overall appearance indicates that positive and negative residuals are not symmetric, and the large slope for the negative residuals indicates that large negative residuals are much stronger than expected.

More diagnostic plots are shown in the supporting information for the interested reader.

The structure model is also affected. For example, the equivalent isotropic atomic displacement parameters *U*_equiv_ from Sim 2 are on average increased by 3.36% compared with those from Sim 1. The increases for individual atoms are up to 10.01% (for C24), followed by 9.10% (C4), 8.51% (C28) and 8.16% (C13). A slight decrease of *U*_equiv_ appears only for the two atoms C14 (−0.19%) and C20 (−0.76%).

## 3. Discussion

The findings show how a simple systematic error that just underestimates all stochastic fluctuations by the same factor and which therefore could be remedied exactly by using a weighting scheme of the type *w* = 1/[*x*^2^ s.u.^2^(*I*_obs_)] – with *x* = 5 in the present case – instead leads to invoking *b* > 0 and to a variety of symmetry-breaking phenomena in the distribution of weighted residuals. The effect is particularly strong for choosing *P* according to equation (2) but would be present, and in fact even stronger, if negative intensity observations were not set to zero in *P*. For the question of why negative intensity observations are set to zero in *P*, the *SHELXL* manual pages offer an explanation: ‘It is possible for the experimental 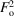 value to be negative because the background is higher than the peak; such negative values are replaced by 0 to avoid possibly dividing by a very small or even negative number in the expression for *w*.’ (https://shelx.uni-goettingen.de/shelxl_html.php). If, for just one moment, negative values for *I*_obs_ in *P* are admitted, negative values in *w* may appear solely through the term *bP* as all other terms are positive or zero. For weak intensities the quadratic term (*aP*) with 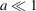 is ignored and we get *w* = 1/(s.u.^2^ + *bP*). Now *b* = 0 holds for a refinement without systematic errors; therefore, systematic errors are needed to invoke *b* > 0. The weight *w* might become negative only for large values of *b*. Large values of *b* appear, for example, when the s.u.(*I*_obs_) are severely underestimated. In short: zero or negative weights indicate a severe error that needs to be removed in order to proceed with the structure refinement. Such a severe error might need reintegration of the raw data with different parameter settings or other substantial changes.

The choice to suppress negative intensity observations in equation (2) allows one to proceed with the refinement despite fundamental errors such as severely underestimated s.u.(*I*_obs_) for the weak data. The price to pay for this is *b* ≫ 0, a distorted residual distribution with large negative residuals and suppressed positive residuals for weak intensities, which together lead to a shift of the mean value of the residuals to negative values and to corresponding changes in the model parameters like the atomic displacement parameters. The topic is even more delicate as the default OMIT command in *SHELXL* suppresses just these large negative residuals that drive the weighted agreement factor, such that two different suppression techniques cut off large residuals from the residual sum: the large positive residuals are cut off ‘from above’ by down-weighting these [see Fig. 1 ▸(*g*)] and the large negative residuals are cut off ‘from below’ (see the supporting information for a visualization) by the default OMIT command. In the present case, using the default OMIT command leads not only to a substantially lower agreement factor and a substantially lower GoF as discussed above but also to a lower value of the weighting scheme parameter *b* = 8.7552 compared with a refinement with the OMIT −100 command. Paying exclusive attention to formal values like *wR*(*F*^2^), GoF, *betc*., it appears as if the weighting scheme and OMIT default settings help to create higher data quality; however, in reality they disguise a simple error that could be easily removed. Such a default OMIT setting is rather specific to *SHELXL* and not implemented in other software packages like *JANA* or *olex.refine*. In the personal view of the author, the default value should be chosen so as to include all reflections.

It is well known that the s.u.(*I*_obs_) values are often underestimated. This is also reflected in the abundant use of the *SHELXL*-like weighting scheme in published datasets with non-zero parameter values: from a sample of 9606 light-atom structures containing exclusively C, O, N and H atoms (to avoid problems with absorption) downloaded from the Crystallography Open Database (Gražulis *et al.*, 2009 ▸), only three structures showed a weighting-scheme parameter of *a* = 0 (COD 2230560, COD 2230904, COD 2016360). One of these three also shows *b* = 0. The use of statistical weights is thus very rare. A total of 6745 datasets (>70%) were processed with one of several releases of *SADABS*; the rest were processed with software by Stoe & Cie, Agilent, Oxford Diffraction and others. In total, a minority of 2039 crystal structures (<22%) were refined with *b* = 0.

There is usually no effort made to clarify the reasons for the need to apply a weighting scheme in standard structure determinations. Inadequacies or errors in the structure model could be such a reason; however, too small s.u.(*I*_obs_) could equally well be a reason. Because no effort is made to discriminate between these cases, it is also not known how large the fraction of datasets is that suffer mainly from severe underestimation of (at least a part of) the s.u.(*I*_obs_) values.

Although the error simulation with fivefold underestimation of s.u.(*I*_obs_) may appear exaggerated to the reader, this example serves just to demonstrate that a bias is induced by a *SHELXL*-type weighting scheme if it is applied in a situation where in particular the s.u.(*I*_obs_) of the weak reflections are underestimated. This poses a methodological problem that persists even in cases where numerical values are not strongly affected.

Blessing (1987 ▸) shows that the variance of the observed intensities might be underestimated by an order of magnitude if the variance of the mean value is employed (rather, the population variance; the difference is that the variance of the mean value is smaller by a factor 1/*n*) for a reflection with redundancy *n* = 100. This is caused by correlations between different reflections introduced by time- and frame-scaling and other data-processing steps, with the effect that the measurements of different reflections are not statistically independent anymore. The loss of statistical independence is also stressed by Sheldrick (2015 ▸): ‘It could be argued that all reflection intensities are independent measurements, and this was approximately true for unscaled data from point detectors before the introduction of focusing optics. However, it is now standard practice to scale the data so that equivalent reflections (usually including Friedel opposites) become more equal, in order to correct for absorption and differences in the effective crystal volume irradiated, and then the equivalent reflections can no longer be regarded as independent observations.’

This error of underestimated s.u.(*I*_obs_) is, to the best knowledge of the author, the first non-trivial example of a systematic error that leads to a negative shift of the residuals. Trivial examples would be a missing extinction correction or unrecognized shadowing by the beam stop and its support, as well as detector saturation. Examples for systematic errors that may lead to a positive shift of the residuals are undetected twinning and unmodelled disorder, as well as low energy contamination and higher harmonics (Domagala *et al.*, 2023 ▸). The (significance of the) deviation of the weighted residuals from zero seems to develop into a good and rather robust overall indicator of systematic errors in diffraction data, similar to high or low blood pressure or high temperature in humans for overall health condition.

## 4. Open questions and final remarks

It was not possible to cover the important question of how the model parameters are affected by systematically underestimated s.u.(*I*_obs_). From the above given information it seems that the atomic displacement parameters are affected the most, but this deserves a more thorough investigation with a variety of structures, which is out of the scope of this work. It was also not possible to address the important question of how other weighting-scheme types like, for example, Chebychev polynomials (Carruthers & Watkin, 1979 ▸) react to underestimated s.u.(*I*_obs_). What conclusions should be drawn? In the definition of *P*, negative intensity observations need to be treated like positive ones in order to fully conform to equation (1). This will increase the bias (as given by the negative shift of the weighted residuals) as the absolute negative residuals will become even larger. This, in turn, may bring the whole concept of equation (1) into question. Another consequence must be to include all reflections in the refinement by setting the default of OMIT to a large negative number, like −100 or so, in order to enable detection of this specific error. The detection of this specific systematic error of underestimated s.u.(*I*_obs_) is hampered or even prevented by (i) the commonly accepted default OMIT value and (ii) the choice of *P* according to equation (2). The IUCr as a science organizing body as well as the crystallographic journals and data banks may need to motivate authors to do that by giving clear rules. Using the OMIT default value *s* = −2 in combination with the error of underestimated s.u.(*I*_obs_) conceals the error and is therefore, to say the least, at the border of massaging the data-quality metrics like GoF and *wR*(*F*^2^). Replacing the observed intensities, whether negative or not, with an idealized value is an unjustified manipulation of the observed data and should be avoided. Whenever large values like *b* > 1 appear it might be worth checking for too small s.u.(*I*_obs_). Standardized protocols for this purpose need to be developed. Taking the findings from this work, checking for 〈ζ〉/σ(〈ζ〉) < −3 and simultaneously (*#*ζ_+_ − *#*ζ_−_)/(*N*_obs_)^1/2^ > +3 in combination with *b* ≫ 1 could be a valuable hint. Finally, some more research may be needed to find out whether or not bias is really reduced in practical applications of a weighting scheme that conforms to equation (1), as in the current example this is clearly not the case. There may be other examples where bias is not reduced but increased instead, and *vice versa*: there may exist examples in which bias is reduced in practical applications. It may be necessary to develop criteria to discriminate between these cases. The derivation of equation (1) by Wilson (1976 ▸), as beautiful and brilliant as it is, is based on limiting assumptions and lacks practical-application examples, which poses a risk of detachment from real-world cases.

In general, all authors of crystallographic structures should be motivated to discuss or at least comment on the underlying causes for non-zero weighting-scheme parameter values and, in particular, for large weighting-scheme parameter values of *a* ≥ 0.1 and *b* ≥ 1 in order to learn from it and to communicate the findings to fellow scientists instead of accepting it unquestioned. Even if the comment is something like ‘Cause unknown’, this will over time get attention from fellow scientists and some eager young scientists may tackle this problem. This will improve the data quality in general and may even lead to helpful and surprising insights.

## Supplementary Material

Supporting information. DOI: 10.1107/S1600576724011889/uz5019sup1.pdf

## Figures and Tables

**Table 1:** Crystallographic values for a refinement against the original observed data from Shraddha *et al.* (2020 ▸) with the ‘OMIT −100’ command, for the reference simulation ‘Sim 1’ and for a simulation with deliberately included systematic error (‘Sim 2’) according to equation (5) (with *x* = 5) with two different values for the OMIT command The command OMIT −100 leaves all negative intensity observations unchanged, while the command OMIT −2 replaces all reflections 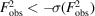 by 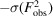.

*hkl* input data	*a*	*b*	*wR*(*F*^2^)	GoF	OMIT
Original	0.0320	1.6958	13.47	1.08	−100
Sim 1	0.0017	0.0047	3.85	1.04	−100
Sim 2	0.0000	13.6794	37.23	1.77	−100
Sim 2	0.0226	8.7552	22.53	1.19	−2†

† The OMIT default value.

**Table 2:** Residual descriptors describing the reference simulation (Sim 1) and the simulation contaminated by a systematic error (Sim 2) with the true noise in the reflections being magnified by a factor of 5 compared with the formal value Column 2: mean weighted residual, 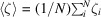. Column 3: significance of deviation of 〈ζ〉 from zero with σ(〈ζ〉) = [var(ζ)/*N*]^1/2^ and population variance 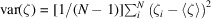. Column 4: mean value of positive residuals. Column 5: mean value of absolute negative residuals. Column 6: mean value of squared positive residuals. Column 7: mean value of squared negative residuals. Columns 8 and 9: integer number of positive and negative residuals. Column 10: ratio of number of positive and negative residuals. Column 11: a measure of the significance of excess residuals when a random walk with 50% probability for positive and for negative steps is assumed. For more on the definitions of these metrics, see *e.g.* Domagala *et al.* (2023 ▸) and Henn (2019 ▸).

	〈ζ〉	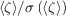	〈ζ_+_〉	〈|ζ_−_|〉	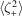	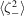	*#*ζ_+_	*#*ζ_−_	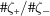	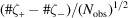
Sim 1	0.00	−0.12	0.80 ± 0.04	0.80 ± 0.04	1.02 ± 0.09	1.00 ± 0.09	2390	2417	0.99	−0.39
Sim 2	−0.20	−8.00	0.83 ± 0.04	1.33 ± 0.11	1.15 ± 0.11	4.93 ± 0.91	2517	2290	1.10	3.27

## Footnotes

1 This is invoked because the variance of the weighted residuals for the weak intensities has increased.
