# Supplementary material for: Bias caused by a popular weighting scheme
Source: J Appl Crystallogr. 2025 Feb 1;58(Pt 1):283–9. doi: 10.1107/S1600576724011889 (PMC11798521; doi:10.1107/S1600576724011889)
Supplement: Supplementary file 1 [file j-58-00283-sup1.pdf]

JOURNAL OF APPLIED CRYSTALLOGRAPHY

Volume 58 (2025)

Supporting information for article:

Bias caused by a popular weighting scheme

Julian Henn

Supplementary Material to

“Bias caused by a popular weighting scheme”

by Julian Henn

The supplementary material contains:

- plots of the weighted residuals sorted in ascending order of Ic, sigma, Ic/sigma and sin theta/lambda for Sim 1 and Sim 2

- a graphic comparing Ueq for all atoms of a test structure from refinements with different values for OMIT command: (i) “OMIT = -100”: includes all reflections; (ii) “OMIT = -2”: SHELXL default value; *replaces* negative intensities by -sigma(Iobs) if they are smaller than Iobs/sigma(Iobs) < -1. This results in a limiting value for the significance of the observed intensities being Iobs/sigma(Iobs) = -1.
For both refinements the same hkl input file and instruction file (model) was used.
For information about the OMIT command see
https://shelx.uni-goettingen.de/shelxl_html.php#OMIT

Table 1: Weighted residuals plotted in rank order of Icalc (A), sigma (B), Icalc/sigma (C) and sin theta/lambda (D) for Sim 1 and Sim 2. Note how the weighted residuals are artificially limited from above and from below in field (3 B) and how this is best visible in just this plot.

	Icalc	Sigma = 1/sqrt(w)	Icalc/sigma	Sin theta/ lambda
	**A**	**B**	**C**	**D**
	Sim 1 (OMIT -100):			
**1**	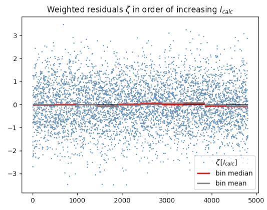	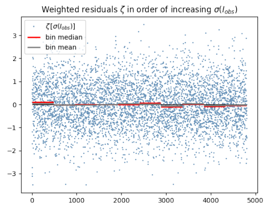	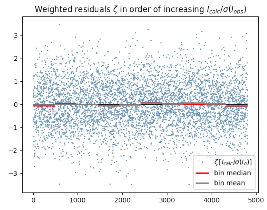	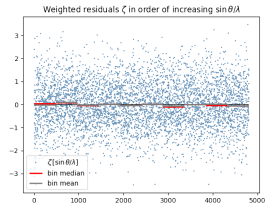
	Sim 2 (OMIT -100):			
**2**	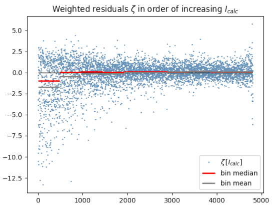	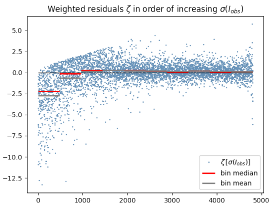	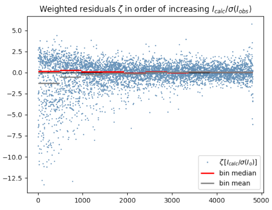	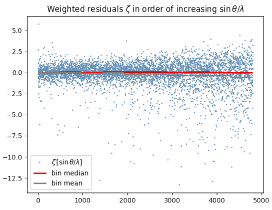
	Sim 2 (OMIT -2)			
**3**	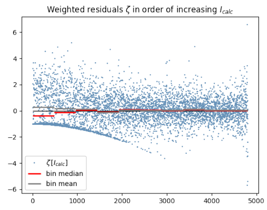	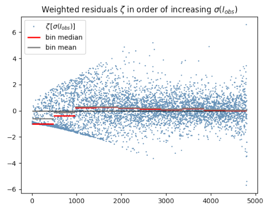	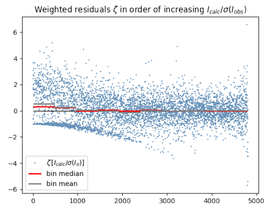	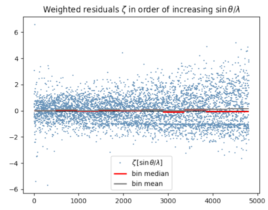

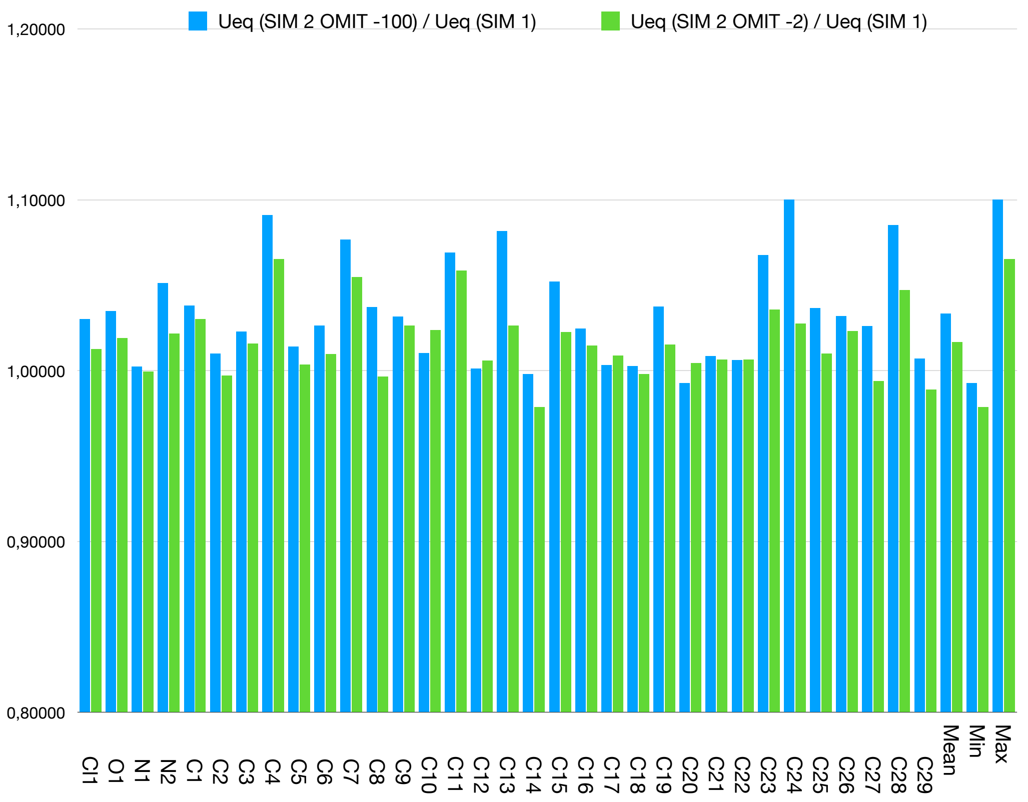

Fig. 1: Ratio of Ueq from simulations for all atoms. A ratio of 1.000 corresponds to the true value from the error-free simulation. The blue bars give the ratio for Ueq from a refinement with systematic error of 5-fold too small formal s.u.(Iobs) values in combination with a refinement with “OMIT -100”. This corresponds to including all negative intensity observations in the refinement. It leads to an average increase of the Ueq of 3.36%. The green bars give the ratio after refinement with the default value “OMIT -2”. In both refinements the same hkl input file and model (ins file) were used. “OMIT -2” leads to an increase in Ueq of 1.66% on average (see the 3rd last pairs of bars on the far right hand side of the plot).

The effect of this specific error of having 5 times larger true physical noise as compared to the formal s.u.(Iobs) values is to increase the Ueq indeed on average by an amount of 3.36% in the present case. The atoms are affected differently, the largest increase is for atoms C24 (+10.01%), C4 (9,10%), C28 (+8.51%), and C13 (+8.16%). For 2 atoms (C14: -0.19%; C20: -0.76%) is the change slightly negative.

Application of the default OMIT command (s = -2) disguises these consequences: The average increase of Ueq is only +1.66% with the largest increases for atoms C4 (+6.54%), C11 (5.85%) and C7 (+5.48%) and the largest decreases for atoms C14 (-2.14%) and C29 (-1,12%)
